# Influenza and pertussis vaccination in pregnancy: Portrayal in online media articles and perceptions of pregnant women and healthcare professionals

**DOI:** 10.1016/j.vaccine.2018.10.092

**Published:** 2018-11-29

**Authors:** Christopher R Wilcox, Kathryn Bottrell, Pauline Paterson, William S Schulz, Tushna Vandrevala, Heidi J Larson, Christine E Jones

**Affiliations:** a NIHR Clinical Research Facility, University Hospital Southampton NHS Foundation Trust, Southampton, UK; b Paediatric Infectious Diseases Research Group, St George's, University of London, UK; c Department of Infectious Disease Epidemiology, London School of Hygiene & Tropical Medicine, London, UK; d Department of Psychology, Kingston University, London, UK; e Faculty of Medicine and Institute for Life Sciences, University of Southampton and University Hospital Southampton NHS Foundation Trust, Southampton, UK

**Keywords:** Vaccination, Pregnancy, Maternal, Media, Confidence, Decision-making

## Abstract

**Introduction:**

Online media may influence women’s decision to undergo vaccination during pregnancy**.** The aims of this mixed-methods study were to: (1) examine the portrayal of maternal vaccination in online media and (2) establish the perceived target of vaccine protection as viewed by pregnant women and maternity healthcare professionals (HCPs).

**Methods:**

Online media articles on maternal vaccination (published July-December 2012 or November 2015-April 2016) were identified through the London School of Hygiene & Tropical Medicine’s Vaccine Confidence Database and thematically analysed. Questionnaires for pregnant women and HCPs were distributed within four English hospitals (July 2017-January 2018).

**Results:**

Of 203 articles identified, 60% related to pertussis vaccination, 33% to influenza and 6% both. The majority positively portrayed vaccination in pregnancy (97%), but inaccurate, negative articles persist which criticize pertussis vaccination’s safety and efficacy. Positively-worded articles about pertussis tended to focus on infant protection and highlight examples of recent cases, whereas positively-worded articles about influenza focused on maternal protection. These themes were reflected in questionnaire responses from 314 pregnant women and 204 HCPs, who perceived pertussis vaccination as protecting the baby, and influenza vaccination as protecting the mother, or mother and baby equally. A minority of the pregnant women surveyed intended to decline influenza (22%) or pertussis (8%) vaccination.

**Conclusions:**

The majority of online articles support pertussis and influenza vaccination during pregnancy. The portrayal of pertussis vaccination as primarily benefiting the child, using real-examples, may influence its higher uptake compared with influenza. This approach should be considered by HCPs when recommending vaccination. HCPs should be prepared to provide advice to women hesitant about vaccination, including addressing any negative media, and consider educational strategies to counteract inaccurate information. Future studies should directly assess the influence of media on vaccine decision-making and establish which media platforms are typically used by pregnant women to gather information.

## 1 Introduction

Vaccination in pregnancy is a safe and effective strategy to protect mothers and young infants from infectious disease at a time when they are particularly vulnerable [1], [2], [3], [4]. A number of vaccines are now routinely offered to pregnant women, including pertussis, influenza and tetanus [5]. Infection with pertussis or influenza can result in adverse events for mother, fetus and infant, including severe respiratory illness and death [6], [7].

Vaccine availability is not a guarantee of vaccine uptake, particularly amongst pregnant women. International campaigns for vaccination have been met with differing levels of acceptance depending on their perceived need and efficacy, as well as safety [8], [9], influencing vaccine confidence [10]. In England, from September 2016 to January 2017 the uptake of influenza vaccination in pregnancy was only 44.9% [11] whilst the uptake of pertussis vaccination was 74.2% [12], however rates vary significantly across different areas of the UK [13], and between different countries [10].

Vaccine confidence is an increasingly important public health issue. The World Health Organization (WHO) Strategic Advisory Group of Experts (SAGE) on immunisation, as well as various national health bodies, have called for improved monitoring of vaccine confidence and further research into the socio-economic determinants of vaccine attitudes [14]. The exponential increase in health-related online resources has also had a significant effect on how patients seek health information globally [15] and can significantly influence patients’ vaccine confidence and decision-making [13].

The aims of this mixed-methods study were therefore: (1) to examine the portrayal of maternal influenza and pertussis vaccination in online media over recent years and consider what influence this may have had on women’s vaccine confidence, and (2) to compare these findings with the perceived target of vaccine protection as viewed by maternity healthcare professional’s (HCPs) and pregnant women, as well as their reported current, or intended, uptake of vaccination.

## 2 Methods

### 2.1 Identification of online media articles and thematic analysis

A search was conducted in the Vaccine Confidence Project’s database at the London School of Hygiene & Tropical Medicine, using the keyword “pregnan*”. This database collects online news articles relating to vaccination (published from any country), as part of a surveillance system to monitor public confidence in vaccination (www.vaccineconfidence.org). News articles were deemed eligible if they related to influenza or pertussis vaccination in pregnancy, and were published in the English language during two different time periods: July to December 2012, or November 2015 to April 2016. Choosing two time periods allowed us to compare how the occurrence of articles on each vaccination, and the media themes within these, had changed over time. The 2012 period was selected as it was during this time that the pertussis vaccine was first routinely introduced for pregnant women in the UK, and the 2015 to 2016 period was the most recent six months of data available to us at the time of the search, and closest to the time when we distributed the questionnaire. The full text was screened of any article considered potentially relevant following the keyword search, and those deemed eligible for inclusion were then coded by theme, and thematically analysed [16]. Coding was conducted by a single author to ensure consistency (KB).

### 2.2 Questionnaire design and development

Two separate questionnaires were developed for pregnant women and maternity HCPs. These were developed with multi-disciplinary input from paediatricians, obstetricians and health psychologists. The questions analysed here were nested within a larger questionnaire focussing on the attitudes of pregnant women and HCPs to both routine vaccination in pregnancy, and to clinical trials of vaccines in pregnancy against respiratory syncytial virus (RSV). The current paper focuses only on the questions related to routinely recommended vaccines (see Supplementary file). Pregnant women were asked whether they had received/were planning to receive influenza and pertussis vaccination, and both pregnant women and HCPs were asked for their opinion as to whether the influenza and pertussis vaccines were given to primarily protect the mother, the baby, or both equally. The study was registered on ClinicalTrials.gov (NCT03096574) and ethical approval was granted (reference 17/LO/0537) prior to recruitment commencing.

### 2.3 Study population and recruitment

The questionnaire for pregnant women was administered to women (aged ≥ 16 years) attending routine pregnancy clinics/wards at four study sites: University Hospital Southampton NHS Foundation Trust, St Georges Healthcare NHS Trust, University Hospitals Bristol NHS Foundation Trust, and Oxford University Hospitals NHS Foundation Trust. The HCP questionnaire was administered to midwives or obstetricians at the same four sites. Recruitment took place between July 2017–January 2018. Pregnant women were recruited in person via opportunistic sampling. HCPs were invited to participate by an email containing a link to an online questionnaire, supported by face-to-face invitations. Participants gave informed consent and questionnaires contained no identifiable information.

### 2.4 Questionnaire data analysis

Data from paper questionnaires were entered at the lead site into iSurvey (www.isurvey.soton.ac.uk). Statistical analysis was performed using GraphPad QuickCalcs (https://www.graphpad.com/quickcalcs). A two-tailed Fisher’s exact test was used to compare frequency of themes in articles. P-values < 0.05 were considered as statistically significant.

## 3 Results

### 3.1 Overview of online media articles

In total, 203 media articles were identified, with 123 articles (61%) originating from the July to December 2012 time period and 80 articles (39%) from the November 2015 to April 2016 time period. Out of the total number of articles, 122 (60%) concerned pertussis vaccination only, 68 (33%) concerned influenza vaccination only, and 13 (6%) concerned both. During 2012, 84/123 (68%) concerned pertussis, 34 (28%) concerned influenza, and five (4%) concerned both. During 2015–2016, 38 (48%) concerned pertussis, 34 (43%) concerned influenza, and eight (10%) concerned both. The majority of the articles were published in the UK (60%), followed by the USA (22%), Australia (8%), Canada (2%), India (2%), New Zealand (2%), Spain (2%), Holland (1%) and South Africa (1%).

### 3.2 Thematic analysis of online media articles

**Fig. 1:**
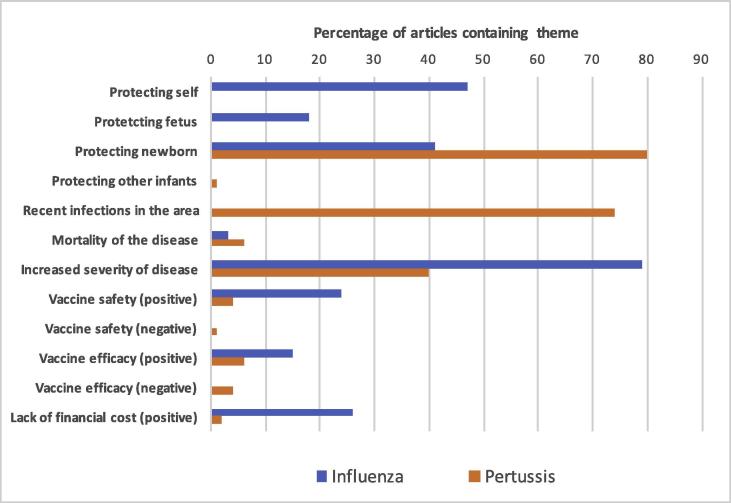
Occurrence of themes in articles regarding influenza and pertussis vaccination in pregnancy between July and December 2012.

**Fig. 2:**
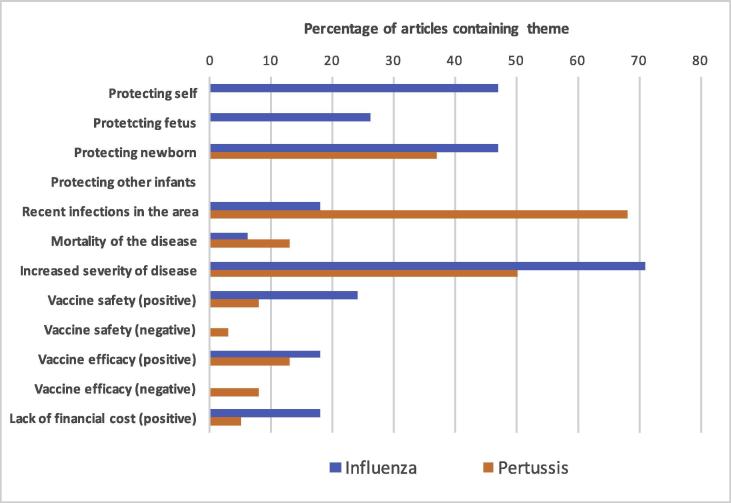
Occurrence of themes in articles regarding influenza and pertussis vaccination in pregnancy between November 2015 and April 2016.

Articles were analysed and 12 themes (10 positive and two negative) were identified (Table 1). Themes were deemed to be ‘positive’ if they were associated with encouraging vaccine use, and ‘negative’ if they were associated with discouraging vaccine use. The overwhelming majority of articles contained positive information, with only seven (3%) containing negative themes, all of which were regarding pertussis vaccination. Fig. 1, Fig. 2 display the themes identified across the articles relating to the influenza and pertussis vaccination across the 2012 and 2015–2016 periods, respectively.

**Table 1:** Themes identified in online media articles relating to influenza and pertussis vaccination in pregnancy, 2012 and 2015–2016.

Themes	Frequency in 2012 influenza articles (N = 34)	Frequency in 2012 pertussis articles (N = 84)	Frequency in 2015–2016 influenza articles (N = 34)	Frequency in 2015–2016 pertussis articles (N = 38)
Protecting self	16 (47%) **	0 (0%) **	16 (47%) **	0 (0%) **
Protecting fetus	6 (18%) **	0 (0%) **	9 (26%) **	0 (0%) **
Protecting newborn	14 (41%) **	67 (80%) **	16 (47%)	14 (37%)
Protecting other infants	0 (0%)	1 (1%)	0 (0%)	0 (0%)
Recent infections in the area	0 (0%) **	62 (74%) **	6 (18%) **	26 (68%) **
Mortality of the disease	1 (3%)	5 (6%)	2 (6%)	5 (13%)
Increased severity of disease	27 (79%) **	34 (40%) **	24 (71%)	19 (50%)
Vaccine safety (positive)	8 (24%) **	3 (4%) **	8 (24%)	3 (8%)
Vaccine safety (negative)	0 (0%)	1 (1%)	0 (0%)	3 (8%)
Vaccine efficacy (positive)	5 (15%)	5 (6%)	6 (18%)	5 (13%)
Vaccine efficacy (negative)	0 (0%)	3 (4%)	0 (0%)	3 (8%)
Lack of financial cost (positive)	9 (26%) **	2 (2%) **	6 (18%)	2 (5%)

Data are N (%).

*p < 0.05.

** p < 0.001.

A significantly higher proportion of articles about influenza vaccination in pregnancy contained the positive theme of “protecting self” compared to those articles about pertussis vaccination in both time periods. The positive theme of “protecting fetus” was also significantly more common in influenza articles in both time periods. In contrast, the positive theme of “protecting newborn” was significantly more common in online media articles about pertussis vaccination during 2012, but during 2015–2016 articles about pertussis vaccination and those about influenza vaccination this difference was no longer observed.

Another positive theme in terms of understanding the importance of the vaccine was “recent infections in the area”. A significantly higher proportion of pertussis articles contained this theme than influenza articles across both the 2012 period and the 2015–2016 period. “Mortality of the disease” was mentioned infrequently in articles about influenza and pertussis vaccination. “Increased severity of disease for the mother”, however, was mentioned more often in influenza articles. The positive theme of “vaccine safety” was identified significantly more often in articles about influenza vaccination compared to pertussis vaccination (Table 1).

Finally, “lack of financial cost” was identified as a positive theme in articles which discussed the availability of vaccines in pregnancy being offered for free as part of routine care. This theme was identified more commonly in articles about influenza vaccination compared to pertussis vaccination across both time periods. “Financial costs” was not identified as a negative theme in any articles.

Seven of the 135 articles mentioning pertussis (5%) contained negative themes. Three of these articles were from the 2012 period, with two criticising the efficacy of the vaccine, and one criticising both its efficacy and safety. Extracts from these articles include:

“More damning evidence of the ineffectiveness of the pertussis vaccine is evident in the current outbreak in Washington State.”

“It's possible that the vaccine isn't as effective as researchers hoped” and “The un-vaccinated people aren't the people getting whooping cough, it's the ones who ARE vaccinated!”

“The truth is that the Tdap shot has never been proven safe for use during pregnancy.”

The remaining four of these articles containing negative themes were from the 2015-2016 period. Three concerned the efficacy of the vaccine, and one concerned its safety. Extracts from these articles include:

“Safety improved but period of protection is less - 6 years now compared to 14 years.”

“There are zero testing results for pregnant women. It has not been demonstrated that the vaccines are safe for pregnancy.”

“There is no evidence yet to support any level of infant immunity from this.”

### 3.3 Questionnaire responses

A total of 321 pregnant women and 204 maternity HCPs completed the questionnaires, across the four sites. Eight questionnaires from pregnant women were excluded due to largely incomplete or illegible responses, leaving 314 pregnant women (97.8%). The characteristics of respondents, including demographic details, are displayed in Table 2. Regarding influenza vaccination, of 310 responses from pregnant women, 118 (38%) had been vaccinated, 123 (40%) were intending to be vaccinated, and 69 (22%) were not intending to receive vaccination. Regarding pertussis vaccination, of 302 responses, 168 (56%) had been vaccinated, 109 (36%) were intending to be vaccinated, and 25 (8.3%) were not intending to receive vaccination.

**Table 2:** Characteristics of the respondents to questionnaires (pregnant women and healthcare professionals).

Characteristic	Pregnant women, N = 314	Healthcare professionals, N = 204
Study site		
A	88 (28%)	45 (22%)
B	77 (25%)	55 (27%)
C	79 (25%)	62 (30%)
D	70 (22%)	42 (21%)

Age (years)		
16–24	34 (11%)	–
25–30	107 (34%)	–
31–35	92 (29%)	–
36–40	58 (19%)	–
41–45	13 (4%)	–
Profession	–	
Obstetrics	–	37 (18%)
Midwifery	–	153 (75%)
No response	–	14 (7%)

Data are N (%).

Both pregnant women and HCPs were also asked whether they thought the influenza and pertussis vaccines were given to primarily protect the mother, the baby, or both equally, see Table 3. Regarding influenza vaccination, out of 300 responses from pregnant women, 58 (19%), 24 (8%) and 218 (73%) responded “the mother”, “the baby” and “both equally”. Of 199 HCP responses, 101 (51%), 5 (3%) and 93 (47%) responded “the mother”, “the baby” and “both equally”, respectively. Regarding pertussis vaccination, out of 303 pregnant women responses, 8 (3%), 172 (57%) and 123 (41%) responded “the mother”, “the baby”, and “both equally”, respectively. Of 199 HCP responses, 4 (2%), 141 (71%) and 54 (27%) responded “the mother”, “the baby” and “both equally”, respectively.

**Table 3:** Response to the question: In your opinion, are the flu and whooping cough vaccines given to pregnant women to primarily protect the mother, the baby, or both equally?

	*Pertussis*			*Influenza*		
	Mother	Baby	Both equally	Mother	Baby	Both equally
Pregnant women (N = 300 or 303)	8 (3%)	172 (57%)	123 (41%)	58(19%)	24 (8%)	218 (73%)
Healthcare professionals (N = 199)	4 (2%)	141 (71%)	54 (27%)	101 (51%)	5 (3%)	93 (47%)

Data are N (%).

## 4 Discussion

To our knowledge, this is the first study to thematically analyse media articles relating specifically to vaccination in pregnancy. It is encouraging that the media surrounding vaccination in pregnancy is dominated by positive messages, yet unfortunately inaccurate negative articles still persist which criticize the safety and efficacy of pertussis vaccination. Articles on pertussis vaccination were more common around the time of its incorporation into routine maternity care during 2012, however more recently, both influenza and pertussis vaccination seem to evoke similar levels of media attention. Articles on pertussis vaccination tended to focus on infant protection and highlight specific examples of recent cases, whereas influenza articles were more focused on maternal protection. These themes were similarly reflected in the questionnaire responses, as respondents tended to perceive the pertussis vaccination as primarily protecting the baby, and the influenza vaccination as protecting the mother, or the mother and baby equally. The reported actual, or intended, uptake of pertussis vaccination was higher than that of influenza (as has been observed in nationally [12], [11] yet fortunately only a minority of the pregnant women surveyed in our study expressed intentions to decline vaccination against influenza (22%) or pertussis (8%).

Differences between the occurrence of these themes may have an influence on vaccine uptake. Previous studies indicate that mothers value protecting their newborn more highly than protecting themselves [17], [18], and reading articles on pertussis which convey a high risk of infection for the baby (especially if associated with real-life cases of mortality or considered ‘close-to-home’) may therefore act as a significant facilitator to receive vaccination. In contrast, the results of this study (as well as previous research involving patient interviews and focus groups [17], [19] demonstrate that influenza is still portrayed as protecting the mother, and there may be less incentive to undergo vaccination. Our findings therefore provide further support that framing vaccine information towards the benefits for the child (ideally using specific examples of real cases) may improve vaccine uptake, in line with recent studies which demonstrate that information emphasising the protective benefits for infants is a major motivator for pregnant women to accept vaccination [20] and improve their health behaviors [21]. It may also be worth placing an emphasis on the interconnectedness of health interests during pregnancy (particularly for influenza vaccination) as whilst mothers may not consider themselves at risk, it should be highlighted that any decline in their health could be seriously detrimental for the health of their child.

It is concerning that negative media messages persist with regards to pertussis vaccination, and that the positive theme of “vaccine safety” was rarely identified from pertussis articles. The majority of these negative articles claimed that there was inadequate trial evidence to support claims of the vaccine’s safety and efficacy, despite the growing amount of high-quality evidence from observational and randomised controlled trials [22], [23], [24]**.** Unfortunately, media articles such as these are likely to have a negative impact on vaccine uptake, as misperceptions of possible harm [8], [17], [25] and inadequate vaccine efficacy and need [26], [27] are commonly cited as the primary reasons for vaccine refusal. It is hopeful that over time, as the supportive evidence for vaccination in pregnancy builds, positive media coverage and public confidence will improve.

### 4.1 Implications for clinical practice and research

The media’s portrayal of pertussis vaccination as primarily benefiting the child, using real-life examples, may influence its higher uptake compared with influenza vaccination, and this approach should be considered by HCPs when promoting vaccination. Providers should be aware that vaccine hesitators are most likely to decline influenza vaccination, and should be prepared to discuss what is influencing their hesitancy, including specifically addressing any negative media that they may have come across. This is especially important as encouragement from a familiar HCP has been shown to increase vaccine acceptance by up to 20 times [8], [28]. Successful innovative strategies to educate women and combat negative media have included the use of social media and webcasts [29], [30], smart phone apps (such as MatImms [31] and iBooks [30]and mobile phone text messages (such as Text4baby) [17], [32]. Outside traditional media channels (which seem to generally support vaccination) it is important to be aware of social media and video-sharing sites which contain large communities of users critical of vaccination, as gathering information from these has been associated with lower vaccine uptake [33], [34]. Healthcare providers could therefore consider counteracting these by uploading positive educational material to these forums.

Future qualitative studies should directly assess the relative influence of media on pregnant women’s vaccine decision making together with other factors. They should also establish which platforms pregnant women would typically use and trust when gathering information, and consider how their views might be modified by such information (both positive and negative). Research conducted over a greater span of time from the vaccine confidence database would also provide a more comprehensive overview of the links between vaccine confidence and media themes, and allow us to better identify trends over time. Future projects should also assess non-English language media sources, particularly as previous studies have demonstrated significantly lower vaccine acceptance amongst ethnic minorities compared to those identifying as White British [35], [36].

### 4.2 Limitations

The major limitations to this study were that the media articles and questionnaire responses were taken from different time periods, and the influence of media on the surveyed sample was not directly assessed. Our search was also limited to the English language for ease and accuracy of analysis, yet we appreciate there may be significant differences amongst non-English language media sources. By using the LSHTM Vaccine Confidence Database we were also limited to articles accessible via the Internet, however we should have captured most media sources given that the majority have an internet presence. By distributing our questionnaire at four hospitals in southern England, we attempted to maximize the demographic diversity of our study population, however the responses cannot be taken as representative of all pregnant women and maternity HCPs. Finally, vaccine acceptance was much higher amongst our questionnaire respondents than national reports of vaccine uptake, and this may limit the generalisability of our study findings. All of our respondents were recruited from antenatal clinics in tertiary hospitals, and therefore it is possible that our sample was missing subsets of the general population which could be more -vaccine critical. On the other hand, we relied upon self-reported vaccination status/intention, and this was not verified through audit of medical records, meaning that there is potential reporting bias in our estimations. However recent studies do suggest that self-reported intention correlates well with actual uptake [37], [38].

### 4.3 Conclusions

Vaccination uptake amongst pregnant women remains suboptimal, yet it is encouraging that the majority of traditional media channels support pertussis and influenza vaccination in pregnancy. The media’s portrayal of pertussis vaccination as primarily benefiting the child, using real-life examples, may influence its higher uptake amongst pregnant women compared with influenza vaccination. We suggest that this approach, as well as the interconnectedness of the health of the mother and the health of the baby, should be emphasised by HCPs when recommending vaccination to pregnant women. HCPs should be prepared to provide advice to vaccine hesitators, including specifically addressing any negative media they may have come across, and consider novel educational strategies which may help counteract any inaccurate negative information.
