# Supplementary material for: Influenza and pertussis vaccination in pregnancy: Portrayal in online media articles and perceptions of pregnant women and healthcare professionals
Source: Vaccine. 2018 Nov 29;36(50):7625–31. doi: 10.1016/j.vaccine.2018.10.092 (PMC6263273; doi:10.1016/j.vaccine.2018.10.092)
Supplement: Supplementary Data 1 [file mmc1.docx]

**Supplementary information**

**A. Questions for pregnant women analysed in this study**

**(1) Have you received either of the following vaccines in this pregnancy?**

Flu (influenza) ☐ Yes ☐ No

Whooping cough (pertussis) ☐ Yes ☐ No

**(1a)** **If no, why have you not yet received these vaccines?**

For flu (influenza)? For whooping cough(pertussis)?

I don’t intend to receive the vaccine ☐ ☐

I haven’t been offered the vaccine yet ☐ ☐

I haven’t got round to getting the vaccine yet ☐ ☐

Other (please specify)……………………………………………………………………………………………………………..

**(2) In your opinion, are the flu and whooping cough vaccines given to pregnant women to primarily protect the mother, the baby, or both equally?**

Flu (influenza) ☐ Yourself ☐ Your baby ☐ Both equally

Whooping cough (pertussis) ☐ Yourself ☐ Your baby ☐ Both equally

**(3) How old are you in years?**

- 1. ☐ 25-30 ☐ 31-35 ☐ 36-40 ☐ 41-45 ☐ 46+ ☐

**B. Questions for healthcare professionals analysed in this study**

**(1) In your opinion, are the flu and whooping cough vaccines given to pregnant women to primarily protect the mother, the baby, or both equally?**

Flu (influenza) ☐ Mother ☐ Baby ☐ Both equally

Whooping cough (pertussis) ☐ Mother ☐ Baby ☐ Both equally

**(2 Which healthcare professional group do you belong to?**

☐ Obstetrics

☐ Midwifery

☐ Other (please state) …………………………………………………………………………………………
